# Mechanisms and Applications of Citral’s Antimicrobial Properties in Food Preservation and Pharmaceuticals Formulations

**DOI:** 10.3390/antibiotics12111608

**Published:** 2023-11-09

**Authors:** María Melissa Gutiérrez-Pacheco, Heriberto Torres-Moreno, María Liliana Flores-Lopez, Norma Velázquez Guadarrama, J. Fernando Ayala-Zavala, Luis Alberto Ortega-Ramírez, Julio César López-Romero

**Affiliations:** 1 Departamento de Ciencias de la Salud, Universidad Estatal de Sonora, San Luis Río Colorado 83430, Sonora, Mexico; melissa.gutierrez@ues.mx; 2 Departamento de Ciencias Químico-Biológicas y Agropecuarias, Universidad de Sonora, H. Caborca 83600, Sonora, Mexico; heriberto.torres@unison.mx; 3 Centro de Investigación e Innovación Científica y Tecnológica, Universidad Autónoma de Coahuila, Saltillo 25070, Coahuila, Mexico; lilianaflores@uadec.edu.mx; 4 Laboratorio de Investigación en Enfermedades Infecciosas, Hospital Infantil de México Federico Gómez, Mexico City 06720, Mexico; normave@himfg.edu.mx; 5 Coordinación de Tecnología de Alimentos de Origen Vegetal, Centro de Investigación en Alimentación y Desarrollo, A. C. Carretera Gustavo Astiazarán Rosas No. 46, Colonia la Victoria, Hermosillo 83304, Sonora, Mexico; jayala@ciad.mx

**Keywords:** citral, biological activities, food additive, pharmaceuticals

## Abstract

Citral is a monoterpene constituted by two isomers known as neral and geranial. It is present in different plant sources and recognized as safe (GRAS) by the Food and Drug Administration (FDA). In recent years, investigations have demonstrated that this compound exhibited several biological activities, such as antibacterial, antifungal, antibiofilm, antiparasitic, antiproliferative, anti-inflammatory, and antioxidant properties, by in vitro and in vivo assays. Additionally, when incorporated into different food matrices, citral can reduce the microbial load of pathogenic microorganisms and extend the shelf life. This compound has acceptable drug-likeness properties and does not present any violations of Lipinski’s rules, which could be used for drug development. The above shows that citral could be a compound of interest for developing food additives to extend the shelf life of animal and vegetable origin foods and develop pharmaceutical products.

## 1. Introduction

Citral is a linear monoterpene aldehyde, present in more than 85% of lemongrass essential oils. This compound is also found in a wide diversity of plant leaves and fruits, such as limes, oranges, lemons, tomatoes, myrtle trees, and African basil [1]. Citral is a mixture of two isomers named neral (cis-3,7-dimethyl-2,6-octadien-1-al) and geranial (*trans*-3,7-dimethyl-2,6-octadien-1-al). Typically, commercial compositions between both compounds are complementary, and this mixture ranges from 48 to 52% of each one [2]. Additionally, this compound is generally recognized as safe (GRAS) by the Food and Drug Administration (FDA) and is commonly used as a citrus base flavoring in different products [3].

Citral has exhibited a broad spectrum of biological activities, where it has been described as an effective antimicrobial agent against different Gram-positive and Gram-negative bacteria, fungi, and parasites of clinical relevance [4,5]. Recent studies have shown that citral has an inhibitory effect against planktonic cells and can also affect biofilms produced by a different microorganism of clinical and food relevance [6,7]. Furthermore, other biological activities have also been demonstrated, such as its antiproliferative effect against human and murine cancer cell lines and anti-inflammatory and antihypertensive properties [8,9,10]. In addition, the capability to stabilize synthetic and biological free radicals and reduce activity has also been reported [11,12].

On the other hand, different studies have analyzed the effect of citral as a food additive incorporated into meat products, fish, fruits, vegetables, juices, and bread [13,14,15,16,17]. Overall, it has been observed that citral effectively reduced the growth and proliferation of pathogenic and deteriorative microorganisms and delayed oxidative processes [13,14,15,16]. In turn, citral extended the shelf life of the added foods. These results promote the possibility of the food industry’s effective and safe use of citral based on studies demonstrating its low cytotoxicity [18].

One of the pivotal aspects of citral’s efficacy lies in its diverse mechanisms of antimicrobial action. This review delves into the biochemical and molecular pathways through which citral exerts its antibacterial, antifungal, antiparasite, and antibiofilm effects. Specifically, it explores how citral disrupts cell membrane integrity, inhibits essential enzymes in microbial metabolism, and interferes with quorum sensing, among other mechanisms [6,19,20]. Understanding these modes of action provides insights into citral’s versatility and paves the way for optimizing its use in food preservation and pharmaceutical applications.

Another important aspect is the potential use of citral as a pharmaceutical agent. This use is based on the broad spectrum of biological activities demonstrated and its low cytotoxicity and compliance with Lipinski’s rule of five [9]. These properties suggest that it can be absorbed orally and reach some target molecules, exerting its biological effect and being able to help in the treatment and control of different health conditions. While substantial progress has been made in uncovering the bioactive properties of citral, several gaps in the analyzed information have been found. Key research questions include: How does citral interact with other food components or pharmaceutical agents to enhance or diminish its effectiveness? Are there potential resistance mechanisms that microbes could develop against citral’s actions? What are the long-term effects of citral exposure on human health and the environment? To address these questions, future research could focus on in-depth mechanistic studies, the development of citral-based combination therapies, and long-term safety assessments. Such investigations would expand our understanding of citral and facilitate its sustainable and efficient application in various sectors. Therefore, the present review aims to show the spectrum of biological activities exhibited by citral and its safe application in the food and pharmaceutical industry.

## 2. Characteristics and Biosynthesis

**Figure 1:**
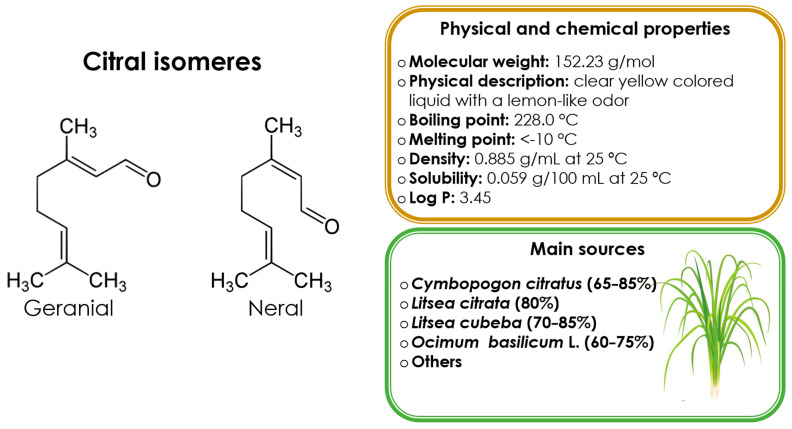
Chemical structures, main sources, and citral’s physical and chemical properties.

Citral is an acyclic monoterpenoid aldehyde consisting of two geometrical isomers, geranial (citral A, *trans*-citral, or the *E*-isomer) and neral (citral B, *cis*-citral, or the *Z*-isomer), in a 3:2 ratio. Citral has the chemical formula C_10_H_16_O, also known as 3,7-dimethylocta-2,6-dienal [21]. Figure 1 shows the structural formula of citral isomers and their main physical and chemical properties [22]. This monoterpene is found in different plants, citrus fruits, herbs, and grasses, being one of the most common essential oils (EOs) of *Cymbopogon* species, mainly East Indian (*C. flexuosus*) and West Indian species (*C. citratus*), commonly known as lemongrass, with a citral content of 70–85% [23,24].

Citral is also present in other plants’ oils, including *Lindera citriodora*, *Calypranthes parriculata*, Petitgrain, *Aloysia citrodora*, lemon ironbark, lemon balm, lime, lemon, orange, lemon tea-tree, among others [25]. Citral content in plant tissues can vary significantly depending on the species and environmental conditions (Table 1). The difference in the quantity can be attributed to the plant´s genetics, climatic factors, seasonal variability, geographic zones, collection period, plant organs, and extraction techniques [26,27].

Citral shows a strong lemon-like aroma and is widely used in cosmetics, fragrances, pharmaceutics, and as a flavor additive in the food industry [39]. Citral shows various biological activities such as antimicrobial [4], antioxidant [40], anticancer [41], anti-inflammatory [42], and others. A growing number of reports published the bioactivities of citral and citral-rich EOs, increasing medicinal and pharmacological significance.

**Figure 2:**
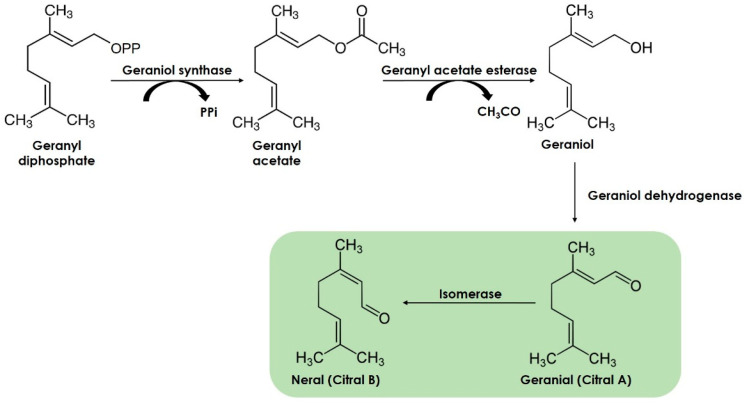
Citral biosynthesis involves multiple transformations from the universal monoterpene precursor GPP.

Citral is biosynthesized from geranyl diphosphate (GPP) molecules after various secondary transformations such as isomerization and acetylation, deacetylation, cyclization, and dehydrogenation (Figure 2) [43].

GPP is considered a universal monoterpene precursor of all monoterpenes and is synthesized by the fusion of two isoprene (C5) units, the isopentenyl diphosphate (IPP) and its isomer dimethylallyl diphosphate (DMAPP) [43]. IPP is the structural unit of all the isoprenoids in plants. It is synthesized by two biochemical pathways localized in different cellular compartments: acetate–mevalonate (acetate–MVA) localized in the cytoplasm and 2C-methylerythritol-4-phosphate (MEP) in the plastids. Both pathways produce different isoprenoids; for one side, the acetate–MVA pathway leads to the synthesis of sesquiterpenes, triterpenes, and sterols, whereas the MEP pathway leads to the formation of monoterpenes, diterpenes, carotenoids, among others [24]. Specifically, it is believed that in lemongrass, the secondary transformations of GPP lead to geraniol, which, after oxidation reactions, leads to citral biosynthesis (Figure 2).

Geraniol is biosynthesized from GPP by the removal of pyrophosphate (PPi) by the geraniol synthase enzyme, which converts GPP to the acetylated geranyl acetate (GA) [43]. Geranyl acetate esterase (GAE) enzyme hydrolyzes GA to geraniol during leaf development in lemongrass [44]. Then, geraniol is further oxidized to citral by a geraniol dehydrogenase enzyme [23].

## 3. Biological Activities

### 3.1. Antibacterial Activity

One of citral’s most relevant biological activities is its antibacterial effect, evidenced against Gram-positive and Gram-negative bacteria (Table 2). In this way, citral obtained from the *Litsea cubeba* (L.) plant was used in the formulation of food packaging films, showing that citral coatings achieved bactericidal effects against *Escherichia coli* (2.1 log) and *Staphylococcus aureus* (4.3 log) at concentrations of 20%. Headspace equilibrium concentrations of 1.8 µg/mL air were found for 20% citral coatings, resulting in a 3.8 log reduction against *E. coli* [45]. Citral, also obtained from *C. flexulosus*, was used against four strains of *Acinetobacter baumannii* with resistance to antimicrobials (MDR = multi-drug resistant), finding that all the tested strains were susceptible to citral with zones of inhibition that varied between 17 and 80 mm. Additionally, citral’s minimum inhibitory concentration (MIC = 0.14% *v*/*v*) and the minimum bactericidal concentration (MBC = 0.3% *v*/*v*) were determined. The ability of citral to inhibit and kill MDR *A. baumannii* highlights its potential for use in treating drug-resistant infections; however, in this study, it is suggested to perform in vitro cytotoxicity studies before human exposure in vivo or ex vivo [46].

*Cronobacter sakazakii* is a foodborne pathogen associated with severe disease and high mortality in newborns and infants. For this reason, Shi, Song [60] evaluated the antimicrobial effect of citral against strains of *C. sakazakii*. They also determined the growth kinetics, membrane potential, and membrane integrity to elucidate the possible antimicrobial mechanism. Citral MICs against *C. sakazakii* strains ranged from 0.27 to 0.54 mg/mL, and citral resulted in a longer lag phase and lower growth rate of *C. sakazakii* compared to the control. Citral affected the cell membrane of *C. sakazakii*, as demonstrated by the decrease in intracellular ATP concentration, the reduction in pH, and the hyperpolarization of the cell membrane. These findings suggest that citral exhibits an antimicrobial effect and could potentially be used to control *C. sakazakii* in food. However, the way it works in food systems, where many other components can interfere with its effectiveness, needs to be tested in future research before its actual application.

The potential of citral on the growth and virulence properties of *Vibrio parahaemolyticus* has been evaluated. Citral showed good antibacterial activity, and the MIC was determined to be 0.125 mg/mL. Citral caused damage to the wall and membrane of bacterial cells based on the morphology observed by electron microscopy. Treatment with citral at sub-inhibitory concentrations caused a decrease in biofilm formation, motility, extracellular production of polysaccharides, and the levels of transcription of genes involved in the production of hemolysin, type III, and VI secretion systems in *V. parahaemolyticus* RIMD2210633. Furthermore, citral inhibited the quorum-sensing genes and the ToxRS system in a concentration-dependent manner [47]. The results strongly suggest that citral could be a promising alternative therapy for combating infections linked to *V. parahaemolyticus*. Its ability to disrupt bacterial virulence factors and inhibit quorum-sensing mechanisms underscores its potential as a valuable option in the fight against infections of this bacteria.

Citral antibacterial activity was evaluated against four clinically isolated strains of bovine mastitis pathogens, including *S. aureus*, *S. agalactiae*, *B. cereus*, and *E. coli*. The results showed that *S. agalactiae* and *B. cereus* are more susceptible to citral than *S. aureus* and *E. coli*. Citral appears to have multiple targets in the bacterial cell, depending on the concentration and number of its components [48]. Another study evaluated the growth kinetics of *L. innocua* and *L. monocytogenes* exposed to citral–carvacrol combinations. The terpene combinations exhibited antibacterial activity against both bacteria, and the effects depended on the concentration of terpenes in the culture medium. When terpene-treated *L. innocua* and *L. monocytogenes* were incubated, significant lag phase and growth rate differences were observed between low and high inoculum concentrations, indicating that the inoculum level should be considered in modeling studies. When bacterial cells were exposed to terpenes, the proportion of sublethal injured cells increased with increasing terpene dose, showing that citral combined with carvacrol used at 25% MIC can control the growth of *Listeria* species. [51]. Possible mechanisms that could lead to synergistic effects between citral and carvacrol are that citral and carvacrol target different sites in the bacterial cell; (i) carvacrol increases the permeability of the outer membrane, which makes it easier for citral to enter the cytoplasm and interact with proteins and nucleic acids; (ii) the combination of the two substances could improve their solubility or bioavailability and thus enhance the antibacterial effect [61]. Citral was also tested against *Salmonella enterica* serovar Typhimurium at sublethal concentrations (MIC = 3.1 mM) and found that citral exerts changes in the fatty acid composition of the membrane, showing limitation of the cyclization of unsaturated fatty acids to cyclopropane fatty acids when cells enter the stationary phase and saturation of the bacterial membrane [53].

The combination of citral with other natural compounds, and in some cases, antibiotics, holds considerable significance in antimicrobial therapy for several reasons. Firstly, it can enhance the antimicrobial efficacy, potentially synergizing the action against a broader spectrum of pathogens. Secondly, this approach may help reduce the risk of antibiotic resistance development, as it can allow for lower antibiotic doses, reducing selective pressure. Thirdly, it offers the potential to simultaneously target multiple mechanisms of bacterial resistance, making it more challenging for bacteria to adapt. Moreover, combining natural compounds like citral with antibiotics can mitigate the adverse side effects associated with high antibiotic doses, thereby improving the overall safety profile of the treatment. In this sense, these combinations can broaden the therapeutic options for infectious diseases, especially in cases of multidrug-resistant pathogens, providing alternative strategies for effective treatment. Nonetheless, it is crucial to conduct rigorous research to assess the safety and efficacy of such combinations before clinical application, considering potential interactions and side effects.

The mechanism of action of citral, a monoterpenic aldehyde, is likely similar to that of other aldehydes. In general, it is known that at low concentrations, an aldehyde can cross-link amino groups in the cell wall and cytoplasm and inhibit enzymes with a thiol group in the cytoplasmic membrane. Citral in high concentrations could target the cytoplasm, causing coagulation and precipitation of the cytoplasmic components. At intermediate concentrations, aldehydes can also cause cytoplasmic clotting [48]. Other authors have mentioned that unsaturated alpha and beta aldehydes have a broad antimicrobial spectrum and show similar activity against Gram-positive and Gram-negative microorganisms [62]. Several authors have reported some of these mechanisms of action against different pathogenic bacteria (Table 3). Geraniol, a monoterpenic alcohol, can affect cytoplasmic membranes and cause intracellular leakage due to the solvation of phospholipids from the membrane. On the other hand, geraniol may cause a loss of structural organization of cytoplasmic membranes for a short period before protein denaturation of the cytoplasm occurs [48].

EOs are being explored as a natural disinfection alternative, and their effectiveness depends mainly on the diverse constituents they may present [63,64]. The EO of the *C. citratus* plant, rich in terpenes such as citral (85%) and geraniol (1.5%), was effective in inhibiting the growth of *E. coli*, *Salmonella* spp., *L. monocytogenes*, and *S. aureus* [64,65,66]. However, several studies evaluated the antimicrobial activity on planktonic cells, that is, against dispersed individual bacterial cells, which differs from the form of bacterial aggregates (biofilms), the true survival mechanism [67]. As observed, citral possesses a remarkable antibacterial activity against different pathogenic bacteria, and in this sense, more studies evaluating their combination with antibiotics are needed. This might provide a more effective treatment for infections caused by antibiotic-resistant strains and address the growing interest in natural and alternative therapies for bacterial infections.

#### Antibiofilm Activity of Citral against Bacteria

The persistence and resistance of some microorganisms to disinfection processes are associated with their ability to form biofilms on surfaces. A biofilm is a sessile bacterial community of cells attached to each other and surfaces [68]. Biofilm adhesion and formation on surfaces are cross-contamination and public health problems [69]. Biofilms can also form on food industry surfaces such as pipes, pumps, and containers used to harvest, transport, and display food [70,71,72]. It has been reported that different bacteria can form biofilms on stainless steel, where insufficient control of surface growth can lead to the detachment of cells responsible for the dispersion of contamination [73]. The foregoing highlights the importance of studying bacterial aggregates’ characteristics to ensure the disinfection processes’ efficiency and consequent assurance of food safety. In this way, citral has shown the capacity to affect biofilm formation (Table 4).

The resistance of *E. coli* O157:H7 to disinfection is associated with its ability to form biofilms, consisting mainly of glucans produced by glucosyltransferases [6]. Citral has shown antibacterial activity against planktonic *E. coli*, showing that citral decreases the production of glucans. However, there is insufficient information on its efficacy and mode of action against *E. coli* biofilms. Furthermore, citral acts as a non-competitive inhibitor of the activity of the enzyme glucosyltransferase. Evidence gathered by coupling analysis indicated that citral could interact with the helix finger of the glucosyltransferase responsible for polymer production [6]. Cellulose synthesis in *E. coli* O157:H7 is regulated by the *bcs* (bacterial cellulose synthesis) genes. The four *bcs* genes are called *bcsA*, *bcsB*, *bcsZ*, and *bcsC* and are organized like an operon. The *bcs* operons are partially regulated by *AgfD*, a thin fimbria that increases the production of cellulose and curli [76]. As mentioned above, cellulose and other extracellular polymeric substances (β-1,6-N-acetyl-D-glucosamine and colanic acid) are key components during the biofilm formation process of *E. coli* O157:H7 [77].

Citral, in combination with carvacrol, demonstrated antibiofilm activity against *C. sakazakii*, showing that the biofilm formation rate of the samples treated with carvacrol (1/2 MIC) and citral (1/2 MIC) for 48 h decreased from 100% to 81.66% and 59.62%, respectively. On the other hand, after treatments with the combinations of carvacrol and citral (1/4 MIC and 1/2 MIC) for 48 h, the rate of biofilm formation was reduced to 33.41% and 6.28%, respectively. Biofilm formation was almost completely suppressed at 37 °C when the combination of citral 1/2 MIC and carvacrol was used. These data revealed that carvacrol and citral had a synergistic inhibitory effect on the biofilm formation of *C. sakazakii*. [55]. Additionally, the authors propose that the genes associated with biofilm formation in *C. sakazakii* were generally downregulated under treatment with the combination of citral, revealing that citral significantly downregulated the expression of *bcsA* and *bcsG* that encode the cellulose biosynthesis operon. Citral may influence the biosynthesis of components associated with EPS and consequently change the structure of biofilm, making it more susceptible. It has been proposed that essential oils may affect the growth of flagella and thereby inhibit the formation of biofilms [78].

Citral was combined with other terpene compounds (carvacrol, thymol, and caprylic acid), and the growth inhibition of *C. sakazakii* in reconstituted infant formulas was evaluated. Results showed that only the combination of citral with carvacrol demonstrated a synergistic effect among the evaluated combinations. This combination had a stronger growth inhibition effect than treatment with citral or carvacrol alone, leading to more portions of cells in the propidium iodide fluorescent region and causing more severe cell damage. Citral and carvacrol also showed potent and promising antibiofilm activity against *C. sakazakii*, which was evaluated by the crystal violet assay, field emission scanning electron microscopy, and qRT-PCR. Gentle heat treatment combined with citral and carvacrol at a concentration of 2 or 3 MIC completely eradicated *C. sakazakii* in the reconstituted infant formula. Furthermore, the combination at 2 MIC had no significant effect on the color and aroma scores of the reconstituted infant formulas [55].

In a similar study, Shi, Sun [75] investigated the antibiofilm efficacy of citral on *C. sakazakii* ATCC 29544. Bacteria were grown at 12 °C and 25 °C for 24, 48, and 72 h on microtiter plates. The biofilm formation was inhibited by 67.1%, 69.5%, and 70.1% with 225 μM citral after treatment at 25 °C for 24, 48, and 72 h, respectively. Compared with the control, cells treated with citral showed significant and dose-dependent inhibition of biofilm formation of *C. sakazakii* [75]. The authors mention that the enhanced antibiofilm effect of citral at 12 °C could be due to the lower metabolic and growth rates of *C. sakazakii* at this temperature, potentially resulting in a weaker biofilm that is more sensitive to citral. In this sense, studies evaluating the antibiofilm potential of citral must consider the biological variability and factors that might influence biofilm inhibition, such as incubation temperatures, pH, ionic strength, surface adhesion materials, and O_2_ and nutrient concentration.

In a recent study, the antibiofilm potential of citral against MRSA was evaluated, and the possible mode of action was deciphered, finding that citral inhibited biofilm formation by MRSA without affecting growth at 100 μg/mL. Microscopic analysis showed that citral greatly hindered the surface adherence of MRSA. Genetic ontology and String analysis revealed that citral differentially regulates proteins involved in pleiotropic transcriptional repression (*CodY*), cell wall homeostasis (*IsaA*), regulation of exotoxin secretion (*SaeS*), adhesion cell, hemolysis, capsular polysaccharide biosynthesis, and pathogenesis. Gene expression analysis and in vitro assays further validated the alteration in the synthesis of slime, hemolysin, lipase, staphyloxanthin, and oxidative susceptibility [7]. MRSA is the main pathogen in biomaterial-associated infections and is well-known to form biofilms in foreign materials. For this reason, Valliammai, Selvaraj [74] evaluated the synergistic antibiofilm activity of citral in combination with thymol to inhibit the adhesion of MRSA to titanium surfaces. The antibiofilm coating effectively inhibited MRSA adhesion in vitro, and the antibiofilm activity of the coating was not affected by plasma conditioning. Furthermore, the antibiofilm coating was neither hemolytic nor toxic to peripheral blood mononuclear cells.

Citral was evaluated against mature biofilms of *S. aureus* SC-01, *L. monocytogenes* EGD-e, or *E. coli* MG1655 in a range of 500–2000 μL/L. The MICs were 200 mL/L for *L. monocytogenes* and 500 mL/L of citral for *S. aureus* SC-01 and *E. coli*, and the concentration of 500 mL/L of citral was selected to be tested against mature biofilms of 72 h of incubation. Citral at 45 °C for 60 min reduced more than five logarithmic cycles of sessile cells that are part of mature biofilms of the three species. These results demonstrate the great potential of citral present in EOs for eradicating biofilms formed by foodborne pathogenic microorganisms [50]. In another study, citral inhibited the biofilm formation of *S. aureus* strains. At an undergrowth inhibitory concentration of 0.25 µL/mL, a 46.5% inhibition on biofilm formation of the clinical *S. aureus* isolate was observed, while the *S. aureus* strain DMST 4745 showed 74.6% of the biofilm formations. This inhibitory effect increased with the increasing concentration of the tested agents [48].

Several studies evaluating the antibiofilm potential of natural compounds such as citral measure the biofilm only as biomass and do not consider the effect of these compounds on the cell viability and EPS components. In this sense, a deeper analysis of the impact of this compound on biofilm as a multicomponent system should be considered, as well as their effect on other virulence factors, to have a broad overview of their antipathogenic effect. Additionally, despite the relevant information of studying citral against single-species biofilms to have an approach to the possible mode of action, several investigations have not evaluated its effect against multispecies-biofilms, as commonly found in the natural environments.

### 3.2. Antifungal Activity

**Figure 3:**
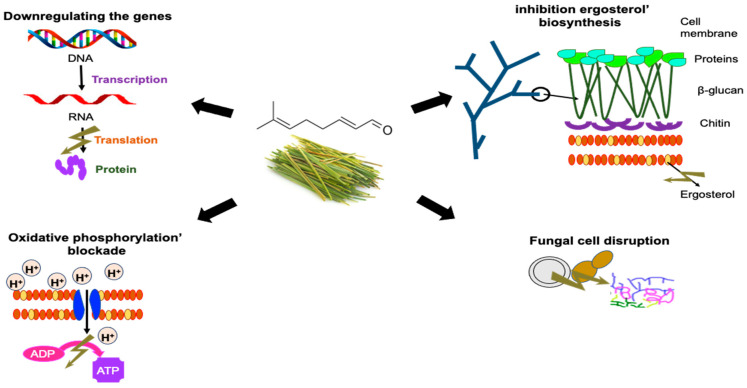
Mechanisms of action of citral as an antifungal agent [54,56,57].

Citral stands out for its broad-spectrum antifungal effect (Table 2), and its mechanisms of action have been extensively studied (Table 5, Figure 3). Recently, *Alternaria alternata*, an important foodborne pathogen, was completely inhibited with a citral dose of 250 µL/L corresponding to the MIC. With a citral dose of ½ MIC, the biosynthesis of the polyketide mycotoxin (alternariol) and its derivative (alternariol monomethyl ether) was also suppressed up to 97% [79]. The transcriptomic profile revealed that citral provoked cell integrity disturbance, specifically by disrupting fungal spores and inhibiting the biosynthesis of ergosterol, a major structural constituent of fungal cell membranes. Interestingly, 41 over-expressed and 84 repressed proteins of *Penicillium digitatum* were identified by isobaric tags for the relative and absolute quantitation technique (iTRAQ) when 1.0 µL/L of citral was applied to the fungus for 30 min [80]. The authors suggested that the citral mechanism involved membrane damage of *P. digitatum* cells and the disruption of oxidative phosphorylation, which is the first way to produce energy in eukaryotic cells. In another study reported by Tang, Chen [81], citral also showed inhibitory action by downregulating the genes related to the sporulation and growth of *Aspergillus ochraceus* and *A. flavus*. While on *A. ochraceus*, the synthesis of proteins associated with the mycelial growth was altered, achieving a fungal complete inhibition at 200 µL/L and the reduction of production of mycotoxin ochratoxin A, a 2B-categorized mycotoxin related to cancer in humans, with a dose of 75 µL/L of citral [54]. Given the broad antifungal activity of citral, its potential as an innovative barrier for controlling fungi in agri-food systems is confirmed. However, further research is needed to explore its incorporation into different products. Factors such as vehicles, specific doses, and bioactivity in each system must be considered during the study.

A recent study evaluated the inhibitory effects of citral against several fungal pathogens (*Botryosphaeria dothidea*, *Phomopsis macrospore*, *and Botrytis cinerea*) present in kiwi fruit. In addition, an attempt was made to describe the antifungal mechanism of citral against *B. dothidea*, which is not completely clarified. The antifungal effect was evaluated, determining the inhibitory rate of mycelial growth on potato dextrose agar Petri dishes supplemented with different concentrations of citral. The results showed a potent inhibition on the growth of the hyphae of the three fungi studied with an MIC of 0.4 μL/mL. Citral changed the morphological characteristics of the fungal hyphae of *B. dothidea*, resulting in loss of cell content and distortion of the mycelium as observed in the scanning electron microscopy (SEM) and electrical conductivity studies, respectively. Citral increased membrane permeability, increased extracellular electrical conductivity, and decreased soluble protein content in *B. dothidea*. A reduction in the range of ergosterol levels demonstrated that citral altered the physiology of the cell membrane. Furthermore, higher concentrations of citral decrease the level of enzymes associated with respiration, resulting in the disruption of energy metabolism (Figure 3). Importantly, citral also showed positive effects on fruit quality and reduced the decay rate of kiwis. Postharvest treatment with citral also maintained fruit quality and reduced spoilage [82].

In another study, the antifungal activity of 20 monoterpenes (including citral) was evaluated against the model yeast *Sacchharomyces cerevisiae*, finding that oxygenated monoterpenes exhibited greater fungistatic and fungicidal activities than hydrocarbons and among the most effective oxygenated monoterpenes was citral with an MIC and minimum fungicidal concentration (MFC) of 0.64 mM. Time response experiments showed that selected monoterpenes rapidly reduce the viability of yeast cells in a time- and dose-dependent manner. Furthermore, reduced viability was associated with loss of cell membrane integrity [57]. The same authors determined the mode of action of citral against *S. cerevisiae* using a flow cytometry study, finding evidence that the acute fungicidal activity of citral against *S. cerevisiae* cells involves loss of membrane and cell wall integrity, resulting in dose-dependent apoptotic/necrotic cell death (Figure 3). However, yeast cells that escape this first alteration of the cell membrane, particularly evident in the sublethal concentration, die by metacaspase-mediated apoptosis induced by the accumulation of intracellular reactive oxygen species (ROS) [57].

Citral obtained from the *Litsea* (L.) *cubeba* plant was used in the formulation of food packaging films, showing that citral coatings achieved antifungal effects against *S. cerevisiae* and *A. niger*, showing a reduction of 4.74 and 4.29 log, respectively, at the concentration of 1.8 µg/mL [45]. Citral significantly inhibited fungal growth and mycotoxin production in *A. ochraceus*. Specifically, 75, 125, 150, and 200 μL/L of citral suppressed mycelial growth by 33%, 46%, 50%, and 100%, respectively. Furthermore, 75 μL/L of citral inhibited ochratoxin-A accumulation by 25%. Through proteomic analysis, an attempt was made to elucidate the inhibitory mechanism of citral on mycelial growth and the production of ochratoxin A in subinhibitory concentrations (75 μL/L), and it was revealed that ochratoxin production is a complex process that involves many associated factors related to various processes, including nutrient intake, sterol biosynthesis, ribosome biogenesis, energy metabolism, oxidative stress, and amino acid metabolism (Figure 3). Furthermore, citral at 75 μL/L downregulated ochratoxin biosynthetic genes, including *pks* and *nrps*, but slightly upregulated global regulatory factors *veA*, *velB*, and *laeA* (Figure 3) [54].

The synergistic antifungal effect of citral and eugenol on *P. roqueforti* was evaluated. The determination of the reactive oxygen species and the malondialdehyde content indicated that the combination could induce the peroxidation of lipids of the membrane, with the contribution of citral being more significant than that of eugenol. Furthermore, the downregulation of the gene encoding a subunit of NADPH oxidase indicated that the explosion of reactive oxygen species induced by the compounding was mediated by NADPH oxidase (Figure 3). The combination of citral and eugenol disrupted the integrity of the cell membrane and internal structures, resulting in the degradation of cellular contents. The combination induced membrane lipid peroxidation and enhanced the ability to destroy the cell membrane. The combined agents eventually caused cell content leakage and cell death [16]. Citral at 2.0 or 4.0 μL/mL strongly damaged *P. digitatum* mitochondria by causing matrix loss and an increase in irregular mitochondria. The extent of deformation mitochondria increased with increasing citral concentrations, with a decrease in the intracellular ATP content and an increase in the extracellular ATP content. Oxygen consumption showed that citral resulted in an inhibition in the tricarboxylic acid cycle pathway of *P. digitatum* cells, inducing a decrease in the activities of citrate synthetase, isocitrate dehydrogenase, α-ketoglutarate dehydrogenase, succinodehydrogenase, and citric acid content while enhancing the activity of malic dehydrogenase in *P. digitatum* cells [56].

Cai, Hu [58] investigated the antifungal activity and mechanism of citral and other terpenes against *Zygosaccharomyces rouxii*, with citral showing the most potent antifungal effect. The antifungal effect can be attributed to the alteration of the integrity and permeability of the cell membrane, which can cause irreversible damage to the cell wall and membrane. They can also destroy yeast proteins and inhibit their synthesis. The results have indicated that EOs can effectively inhibit the growth of food-related microorganisms, and there is great potential for applying EOs to the food industry. The authors mention that the effect of terpenes on the germination capacity of *Z. rouxii* spores should be studied. On the other hand, the possible antifungal mechanism of citral against *Candida albicans* (a yeast that inhabits the human body in a commensal way and can cause opportunistic or pathogenic infections) has also been investigated. The MIC (64 µg/mL) and MFC (256 µg/mL) of citral required only 4 h of exposure to effectively kill 99.9% of the inoculum. Citral’s mechanism of action did not involve the cell wall or ergosterol (Figure 3). However, in the morphological interference test, the product inhibited the formation of *pseudohyphae* and *chlamydoconidia.*

#### Antibiofilm Activity of Citral against Fungi

In addition to the antifungal effect, an antibiofilm potential is another activity widely reported for citral. In this regard, citral obtained from *Melissa officinalis* effectively inhibited the growth of *C. albicans* (MIC = 500 mg/L). Still, it was also able to reduce the formation of mature biofilms by 50% at a concentration of 1000 mg/L [83]. Similarly, terpenoid exposure prevented *C. albicans* from adhering to polystyrene surfaces. Citral, geraniol, thymol, and carvacrol were metabolic activity studies’ most active adhesion inhibitors. Furthermore, they were moderately effective in inhibiting mature biofilms at <2 mg/mL MIC. The results obtained in 2,3-bis(2-methoxy-4-nitro-5-sulfophenyl)-2H-tetrazolium-5-carboxanilide sodium salt (XTT) test were also confirmed by the crystal violet test, where a >50% reduction in absorbance compared to the control indicated the elimination of the biofilm network due to treatment with these terpenoids [83]. Similarly, the EOs of *C. citratus* and *Syzygium aromaticum* rich in citral and other terpenes showed that most of the *C. albicans* strains tested displayed the formation of moderate to strong biofilms. Preformed *Candida* biofilms showed ≥1024 times increased resistance to antifungal drugs. It was two times for *S. aromaticum*, but no increased tolerance for *C. citratus*. The oils were more active against preformed biofilms compared to amphotericin B and fluconazole. At 0.5× MIC, *C. citratus*, followed by *S. aromaticu*, were most inhibitory against biofilm formation. Light and electron microscopic studies revealed the deformity of three-dimensional structures of biofilms formed in the presence of sub-MICs of *C. citratus* (MIC of oils against planktonic cells ranged from 200 to 360 and 100 to 180 g/mL). The cell membranes appeared to be the target site of compounds in sessile cells, as displayed by SEM observations [84]. In the same way, the essential oil of *L. cubeba* (Lour.) Pers. (contains citral and other terpenes) was effective in inhibiting the formation of biofilms and mature biofilms of different *Candida* species by at least 50%, among these are *C. albicans* (2000 mg/L), *C. glabrata* (2000 mg/L), *C. krusei* (250 mg/L), *C. orthopsilosis* (2000 mg/L), *C. parapsilosis* (1000 mg/L), and *C. tropicalis* (2000 mg/L) [85].

Another widely analyzed microorganism is *C. tropicalis*; it has been demonstrated that citral can inhibit biofilm formation (IC_50_: 64 µg/mL) and eradicate preformed biofilms (EC_50_: 128 µg/mL) of *C. tropicalis*. Another study compared the influence of citral and thymol on *C. tropicalis* and its biofilm and the expression levels of certain antifungal tolerance genes. Interestingly, it was observed that both citral (MIC_50_ = 32 µg/mL) and thymol (MIC_50_ = 16 µg/mL) exhibited significant effectiveness against planktonic cells of *C. tropicalis*. After 24 h (log phase) of biofilm formation, the components were added for preformed biofilm. The biofilm inhibitory concentration value for citral was determined to be 64 µg/mL, while thymol inhibited biofilm formation at a lower concentration of 32 µg/mL [86]. Interestingly, as an antifungal agent, citral targets the cell membrane, whereas thymol targets the cell wall. These were evident by the differential expression of ERG11/CYT450 and CNB1 and sorbitol protection assay against citral and thymol. Exogenous ergosterol binding assay showed that citral does not bind directly to ergosterol. Although both citral and thymol exert similar antifungal activity, different action on the cell wall and membranes depicts their diverse mode of action [86]. The administration of citral on *C. tropicalis* biofilm leads to a fungicidal effect. Furthermore, the differential expression of proteome has revealed twenty-five proteins in *C. tropicalis* biofilm, which were differentially expressed in the presence of citral. Among these, amino acid biosynthesis (*Met6p*, *Gln1p*, *Pha2p*), nucleotide biosynthesis (*Xpt1p*), carbohydrate metabolism (*Eno1p*, *Fba1p*, *Gpm1p*), sterol biosynthesis (*Mvd1p/Erg19p*, *Hem13p*), energy metabolism (*Dnm1p*, *Coa1p*, Ndk1p, Atp2p, Atp4p, Hts1p), oxidative stress (*Hda2p*, *Gre22p*, *Tsa1p*, *Pst2p*, *Sod2p*), and biofilm-specific (*Adh1p*, *Ape1p*, *Gsp1p*) proteins were identified [87]. The overexpression of oxidative stress-related proteins indicates the response of biofilm cells in combating oxidative stress during citral treatment.

Moreover, the upregulation of *Adh1p* is of particular interest because it subsidizes biofilm inhibition through ethanol production as a cellular response. The augmented expression of Mvd1p/Erg19p signifies the effect of citral on ergosterol biosynthesis. The presence of citral has shown an increment in hexosamine and ergosterol components in the extracellular matrix of *C. tropicalis* biofilm. Hence, it is indicated that the cellular response towards citral acts through multifactorial processes [87]. In the same study, the influence of citral and thymol on *C. tropicalis* and its biofilms was compared with the expression levels of certain antifungal tolerance genes. The antifungal and antibiofilm activities of both were studied using XTT reduction assay, field emission scanning electron microscope (FE-SEM), confocal laser scanning microscope (CLSM), and real-time reverse transcription polymerase chain reaction (RT-PCR) analysis. Citral and thymol have damaged the cells with distorted surfaces and less viability. Quantitative real-time PCR analysis showed augmented expression of the cell membrane biosynthesis genes, including ERG11/CYT450 against citral and the cell wall-related tolerance genes involving *CNB1* against thymol, thus depicting their differential mode of actions [86].

### 3.3. Antiproliferative Effect against Cancer Cells

The effect of citral against the proliferation of cancer cells has been extensively reported (Table 6). Studies on colorectal cancer cells showed that citral inhibits the growth of HCT116 and HT29 cell lines in a dose- and time-dependent manner without inducing effect in the non-cancerous colon cells CCD841-CoN [9]. Citral reduces the proliferation of the colon cancer cells Caco-2 notoriously [9]. Mohd Izham, Hussin [88] reported that the nano-emulsifying drug delivery system loading with citral (CIT-SNEDDS) was more effective than citral in inhibiting the proliferation of SW620 colorectal adenocarcinoma cells after 72 h of treatment; however, at short times (24 and 48 h), citral was more effective than CIT-SNEDDS to reduce the growth of this cell line. In HT29 cells, citral was more effective than CIT-SNEDDS in reducing cell proliferation. On the other hand, citral was more effective than the antineoplastic drug Cisplatin in reducing the proliferation of the human stomach cancer cells AGS; shrunken cells and generation of shapeless cells were observed after the treatment of AGS cells with citral, which could be correlated with the activation of cell death by apoptosis, where contraction, cell rounding, and the formation of membrane blebs are characteristic. Furthermore, citral showed less cytotoxicity than Cisplatin against the lung fibroblast non-cancerous MRC-5 [9].

Citral strongly suppresses cell proliferation and induces a cytotoxic effect in B16F10 murine skin melanoma cells. In the same way, citral inhibits the proliferation of SK-MEL-147 and UACC-257 human melanoma cells. On the other hand, HaCaT human non-cancerous skin keratinocytes were more resistant to citral than NIH-3T3 murine non-cancerous fibroblasts. The effect of citral on NIH-3T3 was similar to the observed in B16F10 cancer cells, suggesting that citral exhibits greater selectivity against cancer cells in humans [9].

Citral is one of the major chemical constituents of the essential oil of *Lippia citriodora*. Fitsiou, Karafoulidou [90] reported that citral is more effective than the essential oil of *L. citriodora* in inducing cytotoxicity and reducing the proliferation of HepG2 hepatocellular carcinoma cells, MCF-7 breast adenocarcinoma, THP-1 acute monocytic leukemia, A375 malignant melanoma, and Caco-2 cancer cells. Other research has shown that citral reduces the proliferation of KKU-M213 and HuCCA-1 cholangiocarcinoma cancer cells over time; however, the non-carcinogenic bile duct cell line MMNK-1 was more sensitive than HuCCA-1 cells [9]. Balusamy et al. [94] reported that the effect of citral in inhibiting the cell proliferation of PC3 and PC3-M prostate cancer cells is similar to the effect showed by Cisplatin in these cells. However, Cisplatin was more cytotoxic against MRC-5 cells compared to citral. Morphological changes associated with apoptosis cell death, such as shrinkage and rounding, were observed after treating PC-3 and PC-3M cells with citral. In addition, the antiproliferative effect of citral against PaCa-2 pancreatic tumor cells and DeFew B-lymphoma cells has been reported [9].

In vitro studies have shown that citral inhibits the cancer cells colony formation and migration. Balusamy, Ramani [91] reported that citral affects the total number of colonies and the AGS cells’ migration ability (*p* < 0.001 vs. control). Using Hoechst staining and DNA fragmentation assay, it was observed that citral induces chromatin condensation and the nuclear fragmentation of the cells in a dose-dependent manner, probably through apoptosis induction via ROS generation. Citral significantly reduced (*p* < 0.001 vs. control) the clonogenic formation of PC-3 cancer cells. Upekkhawong [93] reported that citral promotes colony formation in KKU-M213 cells and decreases the survival fraction in HuCCA-1 and MMNK-1. These results did not directly correlate with the observed in vitro probably because the doses used in the clonogenicity tests were lower than the IC_50_ values calculated for the in vitro experiments. On the other hand, citral reduced the colony formation of M624 cells more effectively than HaCaT cells [9].

Some mechanisms of action related to the antiproliferative effect of citral against cancer cells have been described. One of the reported mechanisms is the induction of cellular apoptosis. Apoptosis can be triggered through two main pathways: the intrinsic pathway with direct involvement of the mitochondria and the extrinsic pathway mediated through death receptors. Caspases are a family of proteins responsible for activating and executing apoptosis cell death. Activation of intrinsic apoptosis involves the activation of caspase-9 and caspase-3, while the extrinsic pathway promotes the activation of caspase-8 and caspase-3. Caspase-3 is responsible for diverse biochemical changes in the cell, such as DNA fragmentation and the degradation of cytoskeleton proteins. Diverse stimuli such as ROS can trigger intrinsic pathway activation by modulating Bcl-2 proteins. The translocation of phosphatidylserine from the inner membrane to the outer membrane is another biochemical change in cells that die by apoptosis. This change can be detected by flow cytometry using annexin V-FITC/PI [97].

In HCT116 and HT29 cancer cells, citral induces mitochondrial-mediated apoptosis via activation of p53 and increasing the intracellular levels of ROS. Flow cytometry analysis using annexin V-FITC/PI demonstrated that citral induces apoptosis cell death in a dose- and time-dependent manner. In addition, the capacity of citral to increase the intracellular level of ROS and decrease the GHS levels was detected by flow cytometry. By Western blot analysis, it was observed that citral induces the activation of p53, *Bax*, and caspase-3 proteins, while the expression of *Blc-2* and *Bcl-xL* decreased after the treatment with citral [9]. Balusamy et al. [91] investigated the apoptotic effect of citral in AGS cells. The Hoechst and PI stainings showed that citral induces chromatin condensation, while the ability of citral to cause DNA degradation in this cell line was observed by electrophoresis. The annexin V-FTIC/PI analysis demonstrated that citral is a strongly apoptosis-inductive agent in AGS cells. After the treatment with citral, a high accumulation of AGS cells in early and late apoptosis phases was observed. In addition, the transcriptome analysis showed the capacity of citral to modify the expression of some genes associated with MAPK, NF-κB, p53, and PI3K-Akt signaling pathways that control the cells’ survival, apoptosis, and cell cycle. On the other hand, in Caco-2 and HT-29 cells, citral induces cycle arrest in G2/M [9].

In metastatic B16F10 murine cancer cells, the apoptotic induction capacity of citral was demonstrated by annexin V/PI staining and TUNEL assay, while the effect of citral to induce the formation of autophagic vacuoles was observed with the use of mono dansyl cadaverine staining (MDC). Through the analysis of GSH and malondialdehyde levels, as well as the evaluation of the cytotoxic effect of citral by 3(4,5-dimethylthiazole-2-yl)-2,5-diphenyltetrazolium bromide (MTT) in the presence of specific scavengers of ROS (histidine, trolox, tempol), the capacity of citral to induce oxidative stress in B16F10 was demonstrated. The above was consistent with the ability of citral to cause DNA damage, which was observed through the comet assay. Citral interferes with the activation of some signaling pathways in B16F10; after the treatment, citral decreases the expression of ERK1/2, PI3K, and AKT and increases the expression of p53. Likewise, citral inhibits the nuclear translocation of NF-κB in B16F10 cells [9]. In the same way, Skoda et al. reported that the level of caspase-3 activated was higher in M624 with respect to HaCaT cells, which is associated with the greatest cytotoxic/apoptotic effect of citral on M624 in comparison to HaCaT cells [9].

Citral interferes with lipid metabolism, inhibits colony formation, and induces apoptosis in PC-3 cells. Using Oil Red O staining (OSO), it was observed that this compound inhibits the survival of the cells and induces the expulsion of lipid droplets. The quantitative real-time reverse-transcription PCR and the Western blot analysis showed the capacity of citral to activate the AMPαK pathway and induce the downregulation of crucial genes involved in lipogenesis, such as HMCR, ACC, and SREPB1. Nuclear staining with Hoechst and PI dyes showed that citral alters membrane integrity and decreases cell viability. Citral is a strongly apoptosis-inductive agent in PC-3 cells; the labeling with annexin V-FITC/PI showed that citral induced a high percent of cells in early and late apoptosis phases, which was consistent with the modification of the Bcl-2/BAX expression observed by Western blot [9].

In conclusion, studies show that citral effectively inhibits the proliferation of cancer cells in in vitro models. This effect has been associated with the capacity of this bioactive compound to induce apoptosis and cause cell cycle arrest via induction of ROS production, modification of the expression of the Bcl-2 family proteins and p53, and regulation of MAPK, NF-κB, PI3K-Akt, and AMPαK signaling pathways. However, to consider it as a candidate for the development of cancer therapy, it is necessary to evaluate the effectiveness of this metabolite using in vivo cancer models.

### 3.4. Anti-Inflammatory

The anti-inflammatory potential showed by citral has been previously reported (Table 7). Martins, Selis [10] evaluated the anti-inflammatory activity of citral in male mice infected with *S. aureus*. *This bacterium* is one of the most pathogenic species of the staphylococci group and causes several diseases, such as skin diseases, bacteremia, septic arthritis, and respiratory infections. *S. aureus* triggers inflammation and recruitment of neutrophils, critical responses for pathogen clearance but associated with substantial tissue damage. Results indicated that citral inhibited the expression of NO synthase and some features of acute inflammation, such as monocyte numbers and the gene transcription of the pro-inflammatory cytokine TNF-α. The anti-inflammatory activity of citral in RAW 264.7 cells (mouse macrophages, Abelson murine leukemia virus-induced tumor) in the presence and absence of lipopolysaccharide (LPS) was evaluated by Zielińska, Martins-Gomes [98]. Citral inhibited NO production in 84 and 99% at the lowest (5 μg/mL) and highest tested concentrations (20 μg/mL).

**Figure 4:**
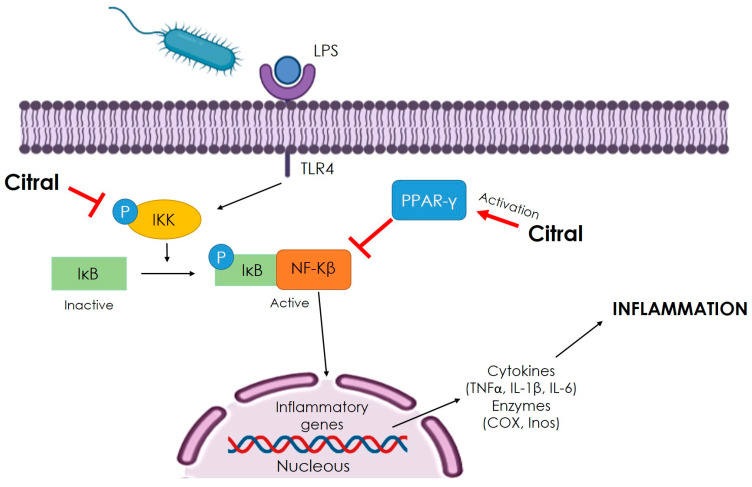
The anti-inflammatory mechanism of citral is attributed to the inhibition of NF-κβ signaling. Citral activates peroxisome proliferator-activated receptor (PPAR-γ) and inhibits IκB phosphorylation, which independently blocks NF-κβ activity with the consequent inhibition of gene expression of the inflammatory mediator [109,110,111,112,113].

In another study, the potential of citral to reduce LPS-induced inflammation was evaluated in a rat model of peritonitis and human umbilical vein endothelial cells (HUVECs). Citral (40 mg/kg) was injected intraperitoneally in rats, whereas the cell line was incubated with 3, 6, and 12 µM of citral for 12 h. Citral treatment reduced the counts of white blood cells and inflammatory cytokines IL-6 and TNF-α in rats. Furthermore, TNF-α and IL-8 expression and NF-κB activation induced by LPS were significantly reduced by citral in HUVECs. Results conclude that the anti-inflammatory activity of citral was attributed to the activation of the PPAR-γ receptor, which attenuates NF-κB activation and inflammatory mediator production [109]. Several other studies demonstrated the anti-inflammatory potential of citral, as shown in Figure 4 [10,104,110,111].

It is well known that citral, like other EOs, shows high volatility, low solubility, and bioavailability that limits its use. Therefore, several studies evaluated the effect of the encapsulation process on their anti-inflammatory potential. Campos, Lima [111] encapsulated citral on β-cyclodextrin (β-CD) and hydroxypropyl-β-cyclodextrin (HP-β-CD) and evaluated their potential to reduce the inflammatory process in mice. For the assay, carrageenan was administrated into the pleural space of mice to induce inflammation, increase leukocyte count, and upregulate the TNF-α production in pleural fluid. It was observed that mice treated with non-encapsulated and encapsulated citral into β-CD and HP-β-CD (0.1 mg/g) significantly reduced total leukocyte counts and TNF-α levels.

Citral showed a potent anti-inflammatory activity, evaluated in many animal and cell models, highlighting those diseases induced by PAMPs, such as LPS of bacteria. Figure 4 resumes some reported mechanisms by which citral exerted anti-inflammatory activity. In general, these include the blockage of NF-κβ signaling, which activates gene expression of inflammatory mediators [109,112].

### 3.5. Antiparasitic

**Figure 5:**
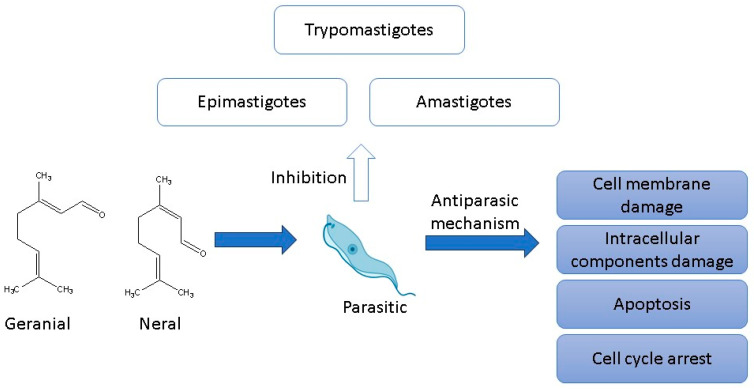
Antiparasitic effect of citral [116,117,118].

Several studies have demonstrated the effect of citral against different stages of trypanosomatids (Figure 5). Santoro, Cardoso [8] observed that citral affected the growth of the epimastigotes and trypomastigotes of *Trypanosoma cruzi* with an IC_50_ of 42 µg/mL and 142 µg/mL after 24 h of incubation, respectively. Furthermore, 50 µg/mL resulted in 100% lysis of trypomastigotes, while 30 µg/mL citral induced a rounded body, mitochondrial swelling, kDNA alterations, and cytoplasmic vacuoles in epimastigotes [8,113,114]. Moreno, Leal [115] also demonstrated that citral exhibited an inhibitory effect against *T. cruzi*, affecting growth in different stages such as epimastigotes (14 µg/mL), trypomastigotes (22 µg/mL), and amastigotes (74 µg/mL). Rojas Armas, Palacios Agüero [116] determined citral’s in vivo antiparasitic effect against *T. cruzi* inoculated in mice. They observed that a dose of 300 mg/kg of citral decreased the proliferation of the parasite at 16, 18, 20, and 22 days post-infection. Furthermore, the same dose of the compound evaluated after 28 days post-infection decreased the amastigote nests in the heart (67.7%) and the infected animal’s inflammatory response (51.7%).

Likewise, Azeredo and Soares [113] evaluated the antiparasitic effect of citral on *Crithidia fasciculata* trypanosomatids and demonstrated that citral has trypanocidal activity. Citral effectively reduced the proliferation of amastigotes and trypomastigotes (IC_50_ and IC_90_ of 76.3 µg/mL and 146 µg/mL, respectively) in this parasite. Santin, dos Santos [117] obtained that citral and *C. citratus* EO (citral major constituent 78%) showed antiparasitic effects against promastigote (IC_50_: 8 and 1.7 µg/mL) and amastigote (IC_50_: 25 and 3.2 µg/mL) of *Leishmania amazonensis*. Also, an effect on intracellular amastigotes was observed at citral and EO concentrations of 5, 10, and 15 µg/mL, reducing the survival index in 0.6 and 47.5% (5 µg/mL), 58.3 and 74.8% (10 µg/mL), and 69.6 and 89.2% (15 µg/mL), respectively.

Similarly, the modes of action of citral and EOs against *L. amazonensis* demonstrated promastigote morphological changes, causing rounded, swollen appearance, flagella damage, mitochondrial swelling, vacuole formation, membrane bleb formation, cell agglomeration, and rupture on membrane plasma. Machado, Pires [118] analyzed the antiparasitic potential of citral and *C. citratus* essential oils against different *Leishmania* strains, observing that both treatments affected *L. infantum* (IC_50_: 42 and 25 µg/mL, respectively), *L. tropica* (IC_50_: 34 and 52 µg/mL, respectively), and *L. major* (IC_50_: 38 and 36 µg/mL, respectively) growth. In addition, it was demonstrated that the antiparasitic effect against *L. infantum* promastigotes nuclear membrane rupture and nuclear chromatin condensation. These treatments produced externalization of phosphatidylserine, loss of mitochondrial membrane potential, and cell cycle arrest in the phase G(0)/G(1), suggesting an effect via apoptosis.

In general, the mechanism of action of citral is related to the induction of microtubule disruption, inhibition of endoplasmic reticulum stress, inhibition of the PIP3/AKT survival pathway, stimulation of oxidative stress, and apoptosis. Furthermore, it is associated with its ability to interact with the cell membranes of parasites, losing its functionality and interacting with membrane components such as flagella. It can also cross cell membranes and interact with intracellular components, causing irreparable damage that promotes proapoptosis and cell cycle arrest [113,115,117].

### 3.6. Antioxidant

**Figure 6:**
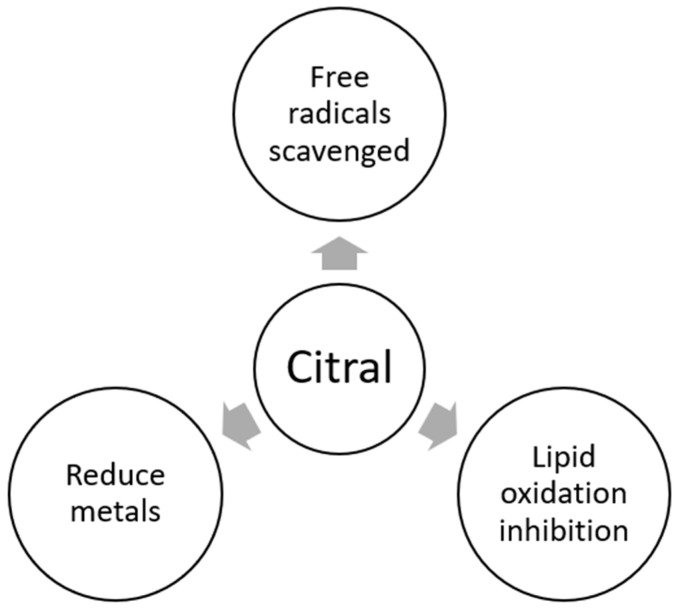
Antioxidant activity of citral [12,123].

The antioxidant activity of citral has been previously explored (Figure 6). In this way, different studies have demonstrated that citral can scavenge the DPPH radical, presenting IC_50_ values between 6.9 and 3700 µg/mL [11,119,120]. A study performed by Xu, Zhu [121] found that citral exhibited the ability to stabilize the DPPH radical (IC_50_: 67.31 mg/mL) and reduce power (concentration of 2 to 10 mg/mL of citral showed an absorbance value between 0.15 and 0.55; high concentration means high absorbance). Bouzenna, Hfaiedh [122] also reported the capacity of citral to stabilize the DPPH radical (EC_50_: 263.33 µg/mL) and reduce metals (FRAP, EC_50_: 125 µg/mL), as well as the capacity to inhibit the oxidation of linoleic acid (β-carotene/linoleate system, inhibition of 71.27%). In the same regard, Guimarães, dasGraças Cardoso [123] tested the capacity of citral to inhibit the DPPH radical and linoleic acid oxidation at a concentration between 5 and 100 µg/L. DPPH results showed that citral had a low antioxidant effect (inhibition lowest to 1.10%). However, the evaluated compound could inhibit linoleic acid oxidation (inhibition between 5.6 and 38%). Wang, Jiang [12] studied the antioxidant potential of citral by several methods. The reduction potential showed a dose-dependent effect, presenting absorbance values between 0.8 and 1.9 at a dose between 0.05 and 0.40 mg/mL. In addition, citral effectively stabilizes the superoxide (IC_50_: 0.67 mg/mL) and hydroxyl (IC_50_: 0.5 mg/mL) radicals. Additionally, citral presented the potential to inhibit lipid peroxidation based on the ferric thiocyanate method, where the analyzed compound at 1 mg/mL reduced linoleic acid oxidation by 47% after 8 days of incubation compared to the control (without citral). The thiobarbituric acid (TBA) method demonstrated that citral reduced malonaldehyde formation by 81%, compared with control (without citral).

The reported results demonstrated that citral may use different mechanisms of action, such as stabilizing free radicals and reducing metals, which is associated with its ability to donate hydrogen, generating more stable radicals due to the presence of double bonds in its structure, promoting the resonance effect [122,124]. Furthermore, citral has shown the ability to effectively stabilize synthetic and biological free radicals, having an essential implication on health since it is well known that oxidative stress plays an important role in the development of chronic degenerative diseases such as cancer, cardiovascular diseases, and neurodegenerative diseases, illnesses that lead the main causes of death worldwide [125,126]. Additionally, it is possible to suggest that citral can act as an antioxidant agent via free radical inhibition, preventing continuous free radical formation and interrupting free radical formation once initiated. On the other hand, the effect of reducing metals is important because metals can induce the formation of free radicals, with a potential oxidative stress generation [127].

## 4. Use as a Possible Food Additive

Citral’s inclusion in the US Environmental Protection Agency (EPA)’s GRAS list as a biopesticide has made it a versatile natural preservative for various food products. This not only extends shelf life but also aligns with consumer demand for healthier, eco-friendly, and clean-label options. Table 8 presents some examples of citral’s applications in food matrices and its effect on food quality. However, citral’s tendency to volatilize could decrease its efficacy or require larger amounts of compound to achieve the desired activity effect. Innovative systems based on nanoemulsions and nanocapsules have emerged to overcome these limitations. These systems protect the active agent and ensure a controlled release, thus prolonging its bioactivity. In a recent study, the antimicrobial activity of citral against *C. albicans* and some bacteria (*E. coli* and *B. cereus*) was enhanced when it was encapsulated in nanostructured lipid carriers (74.8 nm), as citral encapsulates showed lower MIC values than those of the citral emulsion for all microorganisms evaluated [128]. Additionally, Chen et al. [129] reported that liposome–citral nanoencapsulates (105.7–238.0 nm) significantly improved the quality of fresh Shatangju mandarins compared to free citral-treated samples by reducing their weight loss and microbial spoilage after storage at 25 °C and 60–70% relative humidity (RH) for 26 d. Nanoemulsions containing citral have also shown outstanding effects when incorporated into coatings as vehicles, such as the case of the study reported by Machado [130], where alginate-based coatings that include citral nanoemulsions, in an optimal concentration between 0.1–0.5%, were a good barrier against microbial attack, while the quality parameters of the fruit were positively affected (e.g., color and respiration rate) during storage for 12 d at 4 °C and 90% RH.

Furthermore, citral’s antifungal activity has been potentiated when combined with other EOs. For example, a nanoemulsion blending clove (CO) and lemongrass (LGO) oils as eugenol and citral sources effectively disrupted the membrane of the highly invasive *Fusarium oxysporum* f.sp. *lycopersici* fungus. When tested individually, this combination exhibited the lowest MIC value at 3.9 mg/L, compared to 31.3 for CO and 62.5 mg/L for LGO [139]. Similarly, combining citral and eugenol at concentrations of 60 and 170 mg/L resulted in more substantial damage to *P. roqueforti* than when these compounds were used separately, leading to the cell content destruction and, consequently, the death of the fungus [16]. The combination of EOs proves to be a cost-effective strategy, achieving superior results with lower component concentrations. Furthermore, developing nanosystems, such as nanoemulsions and nanoencapsulates, facilitates their incorporation into food products, ensuring prolonged bioactivity.

On the other hand, citral’s antimicrobial properties have been widely studied in food products along with some techniques. High-pressure processing (HPP), a non-thermal technique used to reduce or inactivate certain foodborne microorganisms, often requires high pressures that can compromise food quality. However, recent findings indicate that incorporating citral can reduce the pressure requirements in HPP while improving the inactivation of pathogenic bacteria, such as *E. coli*, commonly found in ground beef and linked to severe intestinal and urinary ailments [15]. With citral concentration at 1% and pressures set at 350 and 400 mPA, a reduction between 3–6 log CFU/g in bacterial concentration is achieved within 24 h and 4 °C. In contrast, to achieve comparable results solely through HPP, a pressure of 500 mPa would be required [15]. This combination has also extended the shelf life of apricot juice. The incorporation of 50 mg/L of citral, coupled with a pressure of 100 mPa reduced the viability of *S. cerevisiae* yeast cells and increased viscosity by enhancing pectin methylesterase activity attributed to HPP [136]. Furthermore, the combination of 3% citral with temperature demonstrated a significant decline in the microbial load of *E. coli* inoculated into ground beef. D-values decreased uniformly across all tested temperatures: 55 °C a 91% decrease; 57.5 °C an 88% decrease; 60 °C a 90% decrease; 55 °C 79% decrease) compared to the control (without citral) [17].

Moreover, there is a correlation between citral concentration and its efficacy. Increasing the concentration has favored its bactericidal effect against *S. Typhimurium* in fish cubes samples. After 4 d at 4 °C, a 3% concentration exhibited higher activity compared to geraniol [13]. Citral also displays selectivity towards the food matrix as an additive. For example, Fisher and Phillips [138] evaluated the effect of citrus EOs (orange, lemon, bergamot) and citral alone on bacteria survival (*Campylobacter jejuni*, *E. coli*, *L. monocytogenes*, *B. cereus*, and *S. aureus*) in cabbage and chicken skin. In cabbage, citral showed a higher inhibition capacity, achieving a 6-log reduction for *S. aureus* and *L. monocytogenes*, while its effect on chicken skin was negligible.

A drawback of citral lies in its sensory attributes; it can exhibit a strong odor and characteristic flavor, poising challenges for direct incorporation into food products. Innovative technologies, including modified atmosphere packaging (MAP), antimicrobial active packaging (AAP), and edible coatings, have been introduced to address this issue. For instance, Muriel-Galet et al. [137] developed an AAP functionalized with citral (7.5% *w*/*w*) and oregano essential oil (7.5% *w*/*w*) within an ethylene-vinyl alcohol copolymer (EVOH) matrix to preserve minimally processed salads. After 8 d, AAP–citral showed a more pronounced reduction in lactic acid bacteria than AAP–oregano essential oil (reductions of 1.19 and 0.44, respectively), with sensory acceptance favoring the salad stored in AAP–citral. In addition, an edible coating based on pectin–citral (2.0% and 0.15%, respectively) effectively extended the shelf life of fresh-cut apples, resulting in less weight loss, improved firmness, and enhanced color retention after 8 d at 4 °C [14]. However, it is worth noting that higher citral concentrations in edible coatings (≥0.5%) can significantly alter the flavor and odor of the product [134].

In the development of edible coatings based on alginate containing a citral–eugenol mixture (0.15 and 0.1%, respectively) and pectin with citral (0.15%), it has been possible to improve the quality of strawberry fruits without compromising their sensory properties (appearance, texture, aroma, and flavor), even after 14 d of storage at 0.5 °C [134].

Citral’s potential enhance food safety and preservation positions it as a valuable additive in the food industry. The current findings in various food matrices strongly suggest that incorporating citral into food production processes can significantly contribute to producing safer and more flavorful food products with extended shelf life, satisfying the industry´s and consumers‘ changing demands.

## 5. Use as Possible Pharmaceutical

In vitro and in vivo studies have shown that citral is a potent agent with many biological activities. However, citral’s ADME-Tox properties (absorption, distribution, metabolism, excretion, and toxicity) have been poorly understood. An in silico study showed that citral isomers (*cis*-citral and *trans*-citral) have acceptable drug-likeness properties and do not present any violations of Lipinski’s rules (molecular weight <500 daltons, Log*Po/w* value <5, <5 hydrogen bond donors, <10 hydrogen bond acceptors) which guarantees their high absorption when administered orally, however, their low solubility in water limits its distribution. Another important factor to consider is that the plasmatic concentration of citral isomers can be reduced due to the high capacity of both compounds to bind to plasmatic proteins [94]. On the other hand, citral isomers could present a high plasma half-life (T1/2) because both compounds do not show an inhibitory effect on CYP2D6. Citral isomers have an acceptable partition coefficient (*cis*-citral Log *Po/w* = 2.74, *trans*-citral Log *Po/w* = 2.71), suggesting both compounds can enter the cell and recognize their therapeutic targets. In addition, the predictive carcinogenicity effect in rodents is variable for the citral isomers (*cis*-citral toxicity (R) = negative, *trans*-citral toxicity (R) = positive); therefore, it is important to consider the concentration of individual isomers during the preclinical evaluations [9].

These results suggest that citral isomers have an acceptable capacity to be absorbed through the gastrointestinal tract and enter the target cells. However, several research studies do not consider the low distribution and bioavailability of this compound, so it is necessary to focus on the design of formulations that guarantee the compound´s good distribution and bioavailability. We also suggest exploring alternative routes of administration beyond the oral route. Finally, knowing the concentration and the stability of the isomers in the pharmaceutical formulations can guarantee reduced short-term and long-term toxicity.

## 6. Conclusions

Citral, a prominent component of various herbs and citrus fruits, offers a remarkable range of biological activities, cementing its promise as an effective natural food preservative and potential pharmaceutical agent. The compound demonstrates broad-spectrum antimicrobial effects, making it particularly valuable in the food industry for extending the shelf life of perishable products. Notably, the FDA has categorized citral as Generally Recognized as Safe (GRAS), emphasizing its potential for widespread and safe application. Importantly, citral’s low cytotoxicity against normal cells and compliance with Lipinski’s rules for drug-likeness suggests that it may also hold promise as a pharmacological agent. This indicates that citral could be an integral compound in drug development, particularly in antimicrobials, anti-inflammatories, and antiproliferative agents. However, while citral’s advantages are compelling, more in-depth studies are crucial for addressing several unanswered questions. For example, how does citral interact with other food components or drugs? Could there be potential mechanisms of microbial resistance against citral? And what are the long-term effects of citral exposure on human health and the environment? Future research should aim to fill these gaps through mechanistic studies, exploring potential synergies in citral-based combination therapies and conducting long-term safety assessments. Ultimately, a more comprehensive understanding of citral’s multi-faceted biological activities and interactions will enable its more effective and sustainable application in the food and pharmaceutical sectors.

## Figures and Tables

**Table 1:** Citral content of different plant species and their extraction methods.

Essential Oil	Plant Material	Citral Content	Collection Site	Extraction Method	Ref.
*Cymbopogon citratus*	Entire plant	48.92%	India	Steam distillation	[28]
	Entire plant	62%	Vietnam	Steam distillation under vacuum	[5]
	Aerial part	74%	-	Solvent-free microwave extraction	[29]
	Dried leaves	80.93%	Brazil	Hydrodistillation	[30]
	Fresh leaves	72.6	South Africa	Hydrodistillation	[31]
*Cymbopogon flexuosus*	Entire plant	43.1%	India	Hydrodistillation	[32]
	Entire plant	74.98%	India	Steam distillation	[28]
*Cymbopogon citrus*	Dried leaves	66.53% and 60.78%	Taiwan	Solar energy extraction and hydrodistillation	[33]
*Lippia alba*	Dried leaves	69%	Brazil	Hydrodistillation	[34]
*Pectis brevipedunculata*	Aerial parts	>91%	Brazil	Hydrodistillation and solid phase microextraction	[35]
*Pectis elongata*	Dried aerial parts	90%	Brazil	Hydrodistillation	[36]
*Citrus limon* var. pompia	Fresh leaves	17.9%	Italy	Steam distillation	[37]
*Lippa citriodora* Kunth	Dried leaves	59.2%	Iran	Hydrodistillation	[38]

**Table 2:** Antibacterial and antifungal activities of citral.

Microorganism	Dose (MIC)	Effect	Ref.
Bacteria			
*V. parahaemolyticus*	0.125 mg/mL	Inhibited bacterial growth, causing damage to bacterial membrane and cell wall.	[47]
*S. aureus DMST 4745* *S. aureus* *S. agalactiae* *B. cereus* *E. coli*	0.62–1.25 μL/mL 0.62–1.25 μL/mL 0.31–0.62 μL/mL 0.15 μL/mL 1.25–2.5 μL/mL	Citral possessed bacteriostatic and bactericidal actions at different concentrations.	[48]
*E. coli* MG1655	300 μL/L	It inactivated at least 2.5 log_10_ cycles of exponentially growing cells in 3 h under aerobic conditions.	[49]
*L. monocytogenes* *S. aureus* *E. coli*	200 µL/mL 500 µL/mL 500 µL/mL	Growth inhibition.	[50]
*L. monocytogenes* *L. innocua*	0.125 mL/mL 0.125 mL/mL	Microbial growth of both *Listeria* species was reduced by almost 2 log_10_ CFU/mL.	[51]
*L. innocua* *L. monocytogenes*	100 µL/mL	Citral in the culture medium of both bacteria provided a reduction of bacitracin from 32 µg/mL to 4 µg/mL, and the colistin changed from 96 and 128 µg/mL for *L. monocytogenes* and *L. innocua*, respectively, to 16 µg/mL, for both species.	[52]
*Salmonella* Typhimurium	3.1 mM	Citral at subinhibitory concentrations (1, 2, and 3 mM) could induce bacterial adaptation and acquire tolerance to inactivation processes.	[53]
Fungi			
*B. dothidea* *P. macrospore* *B. cinerea*	0.2 μL/mL 0.2 μL/mL 0.4 μL/mL	At 0.4 μL/mL, citral entirely inhibited the growth of all the tested fungi. When concentration reached 0.2 μL/mL, citral inhibited the growth of *B. dothidea* best, followed by *P. macrospore* and *B. cinerea.*	[54]
*C. sakazakii*	0.8 mg/mL	Growth inhibition and cell damage.	[55]
	3600 μM	Concentrations below 225 μM (1/16 MIC) exhibited no inhibition against *C. sakazakii* ATCC 29544.	
*Penicillium roqueforti*	0.17 mg/mL	Citral combination with eugenol damaged the cell membrane, caused a collapse of mitochondria, and inhibited energy production.	[16]
*Penicillium digitatum*	2.0 or 4.0 μL/mL	Citral altered the mitochondrial morphology, led to the leakage of ATP, and showed an inhibition of the TCA pathway of *P. digitatum* cells.	[56]
*S. cerevisiae*	2.0 mM	MIC: Results showed that yeast cells treated with 2 mM citral reached a 95% reduction in CFU/mL.	[57]
*Zygosaccharomyces rouxii.*	0.188 μL/mL	The minimum fungicidal concentration was 0.375 μL/mL.	[58]
*Candida albicans*	64 µg/mL	The minimum fungicidal concentration was 256 µg/mL. The MIC and the MFC of citral required only 4 h of exposure to effectively inhibit 99.9% of the inoculum.	[59]
*Aspegillus niger*	0.23 mg/mL	The combination of citral and eugenol had a synergistic inhibitory effect on *A. niger*.	[16]

ATP: adenosine triphosphate; TCA: tricarboxylic acid cycle; MIC: minimal inhibitory concentration; MFC: minimal fungicidal concentration.

**Table 3:** Antibacterial mechanisms of action proposed for citral.

Microorganism	Mechanism	Ref.
*C. sakazakii*	Citral affected the cell membrane of *C. sakazakii*, as demonstrated by the decrease in intracellular ATP concentration, the reduction in pH, and the hyperpolarization of the cell membrane.	[60]
*Salmonella enterica* serovar Typhimurium	Studies on bacteria showed that citral alters the lipid content of *Salmonella enteritidis* cell membranes, increasing the proportion of saturated fatty acids.	[53]
*E. coli* MG1655	Cell death under aerobic conditions was mainly due to oxidative DNA damage and was independent of the tricarboxylic acid cycle, Fenton reaction, and iron availability. Other structures, such as phospholipids, could probably be an important target of citral.	[49]
*V. parahaemolyticus*	Citral caused damage to the wall and membrane of bacterial cells, based on the observation of morphology by electron microscopy. Treatment with citral at sub-inhibitory concentrations caused a decrease in biofilm formation, motility, extracellular production of polysaccharides, and the levels of transcription of genes.	[47]

ATP: adenosine triphosphate.

**Table 4:** Antibiofilm activity of citral against bacteria.

Microorganism	Dose	Effect	Ref.
*Staphylococcus aureus* (MRSA)	100 μg/mL	Citral inhibited the biofilm formation of human severe pathogen MRSA without affecting the growth.	[7]
	25 μg/mL	The percentage of biofilm inhibition by a synergistic combination of citral + thymol was 91%, while 22% and 28% of biofilm inhibition were observed for individual application, respectively.	[74]
*S. aureus* DMST 4745 *S. aureus*	0.25 µL/mL	*S. aureus* DMST 4745 was more susceptible than *S. aureus* clinical isolate to citral, showing biofilm reductions of 74.6 and 46.5%, respectively.	[48]
*C. sakazakii*	0.8 mg/mL	1/2 MIC for 48 h decreased biofilm formation by 59.62%.	[55]
	225 μM	The biofilm formation was inhibited by 67.1%, 69.5%, and 70.1% at the tested concentration after treatment at 25 °C for 24, 48, and 72 h, respectively.	[75]

**Table 5:** Antifungal mechanisms of action proposed for citral.

Microorganism	Mechanism	Ref.
*Saccharomyces cerevisiae* BY4741	Loss of membrane and cell wall integrity results in a typical apoptotic/necrotic cell death. However, yeast cells that escape this first cell membrane disruption, particularly evident in sub-lethal concentration, die by metacaspase-mediated apoptosis induced by the accumulation of intracellular ROS.	[57]
*B. dothidea*	Changes in the morphological characteristics of fungal hyphae, resulting in loss of cell content and distortion of the mycelium. Increase in membrane permeability, with increases in extracellular electrical conductivity and a decrease in soluble protein content. A decrease in the range of ergosterol levels showed that citral altered the physiology of the cell membrane. Reduction in the level of enzymes associated with respiration, resulting in the disruption of energy metabolism.	[54]
*Aspegillus ochraceus*	Citral downregulated ochratoxin biosynthetic genes, including *pks* and *nrps*, but slightly upregulated global regulatory factors *veA*, *velB*, and *laeA.*	[54]
*Aspegillus niger*	Direct damage to the cell membranes of *A. niger* may explain the antimicrobial activity of citral combined with eugenol. Among the two components, eugenol is mainly responsible for the permeability of damaged cell membranes, whereas citral mainly causes membrane lipid peroxidation, which leads to a burst in ROS.	[16]
*Penicillium* *roqueforti.*	The combination of citral and eugenol destroyed the integrity of the cell membrane and internal structures and degraded the cell content. The combination induced membrane lipid peroxidation and promoted the ability to destroy the cell membrane. The combined agents eventually caused leakage of cell contents and, ultimately, cell death.	[16]
*Penicillium* *digitatum*	Citral can affect the mitochondrial morphology and function of *P. digitatum*, inhibiting the respiratory metabolism, decreasing the activities of TCA-related enzymes, and changing the TCA metabolic abilities.	[56]
*Zigosachamomyces rouxii*	The antifungal effect can be attributed to the alteration of the integrity and permeability of the cell membrane, which can cause irreversible damage to the cell wall and membrane. They can also destroy yeast proteins and inhibit their synthesis.	[58]

**Table 6:** Antiproliferative activity of citral.

Compound/ Extract	Doses	Effect	Ref.
Citral	145.32 µg/mL 85.47 µg/mL 52.63 µg/mL	Inhibition of HCT116 cell proliferation (IC_50_: 24, 48, and 72 h)	[89]
Citral	181.21 µg/mL 143.61 µg/mL 91.5 µg/mL	Inhibition of HT29 cell proliferation (IC_50_: 24, 48, and 72 h)	[89]
Citral	3.125–200 µM	Inhibition of CCD841-CoN cell (IC_50_ not detected at 200 μM)	[89]
Citral	3.7 µg/mL	Inhibition of Caco-2 cell proliferation (IC_50_: 72 h)	[90]
CIT-SNEDDS	38.50 µg/mL 23.75 µg/mL 16.50 µg/mL	Inhibition of SW620 cell proliferation (IC_50_: 24, 48, and 72 h)	[88]
CIT-SNEDDS	44.10 µg/mL 36.60 µg/mL 34.10 µg/mL	Inhibition of HT29 cell proliferation (IC_50_: 24, 48, and 72 h)	[88]
Citral	31.25 µg/mL 23.30 µg/mL 22.50 µg/mL	Inhibition of SW620 cell proliferation (IC_50_: 24, 48, and 72 h)	[88]
Citral	28.33 µg/mL 22.00 µg/mL 21.77 µg/mL	Inhibition of HT29 cell proliferation (IC_50_: 24, 48, and 72 h)	[88]
Citral	<25 µg/mL	Inhibition of AGS cell proliferation (IC_50_: 48 h)	[91]
Citral	>75 µg/mL	Inhibition of MRC-5 cell proliferation (IC_50_: 48 h)	
Citral	1.04 µM	Inhibition of B16F10 cell proliferation (IC_50_: 24 h)	[92]
Citral	11.7 µM	Inhibition of SK-MEL-147 cell proliferation (IC_50_: 24 h)	
Citral	13.4 µM	Inhibition of UACC-257 cell proliferation (IC_50_: 24 h)	
Citral	50.3 µM	Inhibition of HaCaT cell proliferation (IC_50_: 24 h)	
Citral	2.5 µM	Inhibition of NIH-3T3 cell proliferation (IC_50_: 72 h)	
Citral	7 µg/mL	Inhibition of HepG2 cell proliferation (IC_50_: 72 h)	[90]
Citral	1.3 µg/mL	Inhibition of MCF-7 cell proliferation (IC_50_: 72 h)	
Citral	71.90 µM 57.11 µM 50.20 µM	Inhibition of KKU-M213 cell proliferation (IC_50_: 24, 48, and 72 h)	[93]
Citral	94.43 µM 75.06 µM 58.92 µM	Inhibition of HuCCA-1 cell proliferation (IC_50_: 24, 48, and 72 h)	[93]
Citral	87.53 72.17 69.22	Inhibition of MMNK-1 cell proliferation (IC_50_: 24, 48, and 72 h)	[93]
Citral	10 µg/mL	Inhibition of PC-3 cell proliferation (IC_50_: 72 h)	[94]
Citral	12.5 µg/mL	Inhibition of PC-3M cell proliferation (IC_50_: 72 h)	[94]
Citral	>75 µg/mL	Inhibition of MRC-5 cell proliferation (IC_50_: 72 h)	[94]
Citral	238 µM	Inhibition of PaCa-2 cell proliferation (IC_50_: 72 h)	[95]
Citral	300 µM	Inhibition of DeFew cell proliferation (IC_50_: 72 h)	[95]
Citral	5, 10, 20, 40 µg/mL	Inhibit colony formation and migration of AGS (96 h)	[91]
Citral	5, 10, 20, 30, 40 µg/mL	Inhibit colony formation and migration PC-3 (96 h)	[94]
Citral	17.5 and 35 µM	Increase the surviving fraction of KKU-M213 in 106.75 and 115.64% (168 h)	[93]
Citral	23.5 and 47 µM	Decrease the surviving fraction of HU-CCA-1 in 76.35 and 57.71% (168 h)	[93]
Citral	24 and 48 µM	Decrease the surviving fraction of MMNK-1 in 98.46 and 85.26% (168 h)	[93]
Citral	0.25, 0.375, 0.50 mM 0.25, 0.375, 0.50 mM	Decrease the clonogenicity of HaCaT in 0.3, 4, and 7% (3 h) Decrease the clonogenicity of HaCaT in 22, 28, and 30% (8 h)	[96]
Citral		Decrease the clonogenicity of M624 in 20, 38, and 50% (3 h)	[96]
Citral	50 µM 100 µM 200 µM	Early apoptosis (17.1%), late apoptosis (3.1%) in HCT116 (24 h) Early apoptosis (14.2%), late apoptosis (15.1%) in HCT116 (24 h) Early apoptosis (26.2%), late apoptosis (25.8%) in HCT116 (24 h)	[89]
Citral	50 µM 100 µM 200 µM	Early apoptosis (22.3%), late apoptosis (16.1%) in HCT116 (48 h) Early apoptosis (26.2%), late apoptosis (24.6%) in HCT116 (48 h) Early apoptosis (32.1%), late apoptosis (37.5%) in HCT116 (48 h)	[89]
Citral	50 µM 100 µM 200 µM	Early apoptosis (6.5%), late apoptosis (3.9%) in HT29 (24 h) Early apoptosis (8.5%), late apoptosis (14.2%) in HT29 (24 h) Early apoptosis (8.4%), late apoptosis (24.9%) in HT29 (24 h)	[89]
Citral	50 µM 100 µM 200 µM	Early apoptosis (14.5%), late apoptosis (7.1%) in HT29 (48 h) Early apoptosis (22.7%), late apoptosis (17.8%) in HT29 (48 h) Early apoptosis (30.5%), late apoptosis (23.5%) in HT29 (48 h)	[89]
Citral	10 and 20 µg/mL	Induce early and late apoptosis in AGS	[91]
Citral	1 µM	Apoptosis induction by annexin V-FITC/PI staining in B16F10 (24 h)	[92]
Citral	0.5, 1, and 2 µM	Apoptosis induction by TUNEL assay in B16F10 (24 h)	[92]
Citral	10 µg/mL 20 µg/mL	Early apoptosis (44.1%), late apoptosis (52.6%) in PC-3 (48 h) Early apoptosis (62.2%), late apoptosis (38.4%) in PC-3 (48 h)	[94]
Citral	50, 100, and 200 µM	Disruption of MMP (19.5, 38.8 and 60.9%) in HCT116 (24 h)	[89]
Citral	50, 100, and 200 µM	Disruption of MMP (34.9, 56.4 and 77.3%) in HCT116 (48 h)	[89]
Citral	50, 100, and 200 µM	Disruption of MMP (20.4, 28.2 and 41.9%) in HT29 (24 h)	[89]
Citral	50, 100, and 200 µM	Disruption of MMP (24.5, 43.9 and 59.9%) in HT29 (24 h)	[89]
Citral	50, 100, and 200 µM	Increase intracellular ROS level (1.26, 2.07, and 3.19 folds) in HCT116 (4 h)	[89]
Citral	50, 100, and 200 µM	Increase intracellular ROS level (1.21, 1.39, and 2.25 folds) in HC29 (4 h)	[89]
Citral	50, 100, and 200 µM	Decrease intracellular GSH level in HCT116 (4 h)	[89]
Citral	50, 100, and 200 µM	Decrease intracellular GSH level in HT29 (4 h)	[89]
Citral	1 µM	Autophagic vacuole induction formation in B16F10 (24 h)	[89]
Citral	0.5, 1, and 2 µM	DNA damage in B16F10 (24 h)	[92]
Citral	2.5 µM	Reduction of malondialdehyde level in B16F10 (24 h)	[92]
Citral	10 and 20 µg/mL	Inhibition of lipid droplet accumulation in PC-3 (48 h)	[94]
Citral	50, 100, and 200 µM	Down-expression of Bcl-2 and Bcl-xL proteins in HCT116 (24 h) High expression of Bax, p53, and caspase-3 proteins in HCT116 (24 h)	[89]
Citral	50, 100, and 200 µM	Down-expression of Bcl-2 and Bcl-xL proteins in HT29 (24 h) High expression of Bax, p53, and caspase-3 proteins in HT29 (24 h)	[89]
Citral	0.5 and 1 µM	Down-expression of ERK1/2, PI3K, AkT in HCT116 (24 h) High expression of p53 in HCT116 (24 h)	[92]
Citral	1 µM	Increase cytoplasmatic NF-κB in B16F10 (24 h)	[92]
Citral	1 µM	Decrease nuclear translocation of NF-κB in B16F10 (24 h)	[92]
Citral	0.25, 0.375, 0.5 mM	Caspase-3 activation in M624 (3 h)	[96]
Citral	0.25, 0.375, 0.5 mM	Caspase-3 activation in HaCaT (3 h)	[96]
Citral	20 µg/mL	Down-expression of HMGR, SREPB1, and ACC proteins in PC-3 (48 h) Up-expression of AMPαK in PC-3 (48 h)	[96]
Citral	5, 10, and 20 µg/mL	Down-expression of BCl-2 in PC-3 (48 h) and high expression of BAX proteins in PC-3 (48 h)	[96]
Citral	Not reported	mRNA upregulate in AGS (48 h): MAPK, Nf-κB, PI3K-Akt, p53, and other signaling pathways. Spliceosoma, apoptosis, and prostate cancer, among others.	[91]
	Not reported	mRNA downregulate in AGS: NF-κB, PI3K-Akt, p53, PPAR, among other signaling pathways. Cell cycle, fatty acid metabolism, and proteoglycans in cancer, among others.	[91]
Citral	5, 10, and 20 µg/mL	Down-expression of *HMCR*, *ACC*, *FASN*, and *SREPB1* mRNAs in PC-3 (48 h)	[94]

CIT-SNEDDS: Nano-emulsifying drug delivery system loading with citral. IC_50_: half-maximal inhibitory concentration.

**Table 7:** Anti-inflammatory activity of citral, its isomers, and citral-rich EOs.

Citral/EO Citral Rich/Constituent	Concentration	Animal/Cell Line Tested	Results	Ref.
Citral	5–100 μg/well	Peritoneal macrophage of male BALB/c mice	50 and 100 μg of citral significantly inhibited IL-1β and IL-10 release and LPS activation. IL-6 production by macrophages significantly decreased at citral concentrations of 5, 10, 25, 50, and 100 μg/well).	[99]
Citral	0.36, 0.15, and 0.06 g/kg	MRSA-infected mice	Citral significantly reduced the levels of TNF-α, IL-6, IL-1β, malondialdehyde, and hydroxyl radicals. Increased superoxide dismutase and glutathione enzyme levels. Reduced the lung inflammatory infiltrates infected by MRSA.	[100]
Citral	300 mg/kg	Diabetes-induced rats	Gene expression of IL-6 and TNF-α in the liver were significantly downregulated.	[101]
Citral	50–300 mg/kg	Paw edema-induced mice	Reversed paw edema formation in mice induced by LPS and zymosan, inducers of TLR4 and TLR2 signaling.	[42]
Citral	300 mg/kg	Eutrophic and obese mice	Citral reduced TNF-α and serum leptin concentration after the LPS challenge. IL6 levels in the hypothalamus of obese mice were reduced.	[102]
Citral	125, 250, and 500 mg/kg	Male Swiss mice	Citral reduced NO production and inhibited neutrophil migration in liver.	[103]
Citral	10, 20, and 40 mg/kg 3, 6, and 12 µg/mL	Mice with LPS-induced acute lung injury Alveolar macrophages	On in vivo LPS-induced acute lung injury, citral reduced TNF-α, IL-6, and IL-1β production. In vitro, citral inhibited the production of TNF-α, IL-6, and IL-1β in alveolar macrophages. The mechanism was associated with PPAR-γ activation.	[104]
Citral, neral, and geranial	66 µM	Murine J774A.1 macrophages	Citral inhibited TNF-α and IL-6. Pure neral inhibited TNF-α secretion by 60–80%, whereas geranial 57–75%. Both neral and geranial reduced IL-6 secretion of LPS-stimulated macrophages and the expression of inflammatory mediators IL-1β, iNOS, COX-2, and NLRP-3.	[105]
Citral-rich fractions of Citrus lemon EO	0.005, 0.01, and 0.02%	Murine macrophage RAW264.7 cell line	Reduced the expression of the pro-inflammatory cytokines TNF-α, IL-1β, and IL-6 in LPS-induced macrophages.	[106]
*Cymbopogon citratus* EO	0.1%	Pre-inflamed human dermal fibroblasts	Significantly inhibited the production of the inflammatory biomarkers: vascular cell adhesion molecule 1 (VCAM-1), interferon gamma-induced protein 10 (IP-10), interferon-inducible T-cell alpha chemoattractant (I-TAC), and monokine induced by gamma interferon (MIG).	[107]
*Myrcia ovata* EO	200 and 300 mg/kg	Male Swiss mice with induced acute inflammation	Reduced leukocyte extravasation and inhibited TNF-α production by 50% and 69% at both concentrations, as well as IL-1β production by 47%.	[108]

**Table 8:** Application of citral in foods.

Food System	Structural Matrix and Dose	Effect	Ref.
Kiwifruit	Citral, 600 µL/L	Extension of postharvest quality by enhancing antioxidant capacity.	[82]
Bread	230 mg/mL each, citral and eugenol	Antifungal activity on *A. niger* and shelf life extension.	[16]
Fruit juices	Citral and vanillin, 100 and 1000 mg/L, respectively, and UV-C treatment	5-log growth reduction of *E. coli*, *L. plantarum*, and *S. cerevisiae*.	[131]
Fresh-cut pineapples	Alginate-based coating containing citral nanoemulsions at 0.1–0.5%	Reduced microbial growth and improved shelf life quality.	[130]
Shatangju Mandarin	Liposome-nanoencapsulated citral at 125 g/L	Shelf life extension and antimicrobial effect on *E. coli*, *B. subtilis*, *S. aureus*, and *P. italicum*.	[129]
Tomato fruit	1-methylcyclopropene and citral, 1.0 µL/L and 5.8 µL, respectively	Suppression of spore germination and mycelia growth of *Botrytis cinerea*.	[132]
Fresh chicken fillets	Kafirin-based films incorporating 1.25% citral and 1.0% quercetin	Antimicrobial activity against total viable count and improvement of quality.	[133]
Ground beef	1% citral and high-pressure homogenization treatment	Promoted inactivation of *E. coli* and improved the high-pressure homogenization treatment.	[15]
Ground beef	1%, 2%, and 3% of citral and temperature (55, 57.5, 60, and 62.5 °C)	The combination of citral and temperature significantly reduced the concentration of *E. coli* at all tested temperatures.	[17]
Fresh-cut apple	Pectin-based coatings enriched with 0.15% citral.	Control of microbial spoilage and preservation of fruit quality.	[14]
Raspberry	Pectin-based coatings enriched with 0.15% citral and 0.1% eugenol	Control of microbial spoilage and preservation of fruit quality.	[134]
Strawberry	Pectin-based coatings enriched with 0.15% citral and alginate-based coatings enriched with 0.15% citral and 0.1% eugenol.	Better sensorial qualities.	[134]
Fresh-cut apples	125 mg/L each, citral and hexanal+2-(E)-hexenal	Inhibition of yeast spoilage and shelf life and quality extension.	[135]
Apricot juice	50 mg/L citral and high-pressure homogenization treatment	Inhibition of *S. cerevisiae* SPA.	[136]
Packaged salad	7.5% of citral in an antimicrobial active bag of EVOH	Reduced the number of lactic acid bacteria.	[137]
Cabbage and chicken skin	0.03 and 0.06% of citral	Antimicrobial activity against *L. monocytogenes* and *S. aureus* in cabbage.	[138]
Fish cubes	0.5–3.0% of citral	Antimicrobial effect against *S. typhimurium*.	[13]

EVOH: Ethylene-vinyl alcohol copolymers.

## Data Availability

Not applicable.
